# Mitofusin-2 Negatively Regulates Melanogenesis by Modulating Mitochondrial ROS Generation

**DOI:** 10.3390/cells11040701

**Published:** 2022-02-16

**Authors:** Jyoti Tanwar, Suman Saurav, Reelina Basu, Jaya Bharti Singh, Anshu Priya, Maitreyee Dutta, Uma Santhanam, Manoj Joshi, Stephen Madison, Archana Singh, Nirmala Nair, Rajesh S. Gokhale, Rajender K. Motiani

**Affiliations:** 1 CSIR-Institute of Genomics and Integrative Biology (IGIB), New Delhi 110025, India; jyoti.tanwar@igib.in (J.T.); reelina.basu@gmail.com (R.B.); anshupriyaap62@gmail.com (A.P.); archana@igib.in (A.S.); rsg@nii.res.in (R.S.G.); 2 Academy of Scientific and Innovative Research (AcSIR), Ghaziabad 201002, India; 3 Laboratory of Calciomics and Systemic Pathophysiology, Regional Centre for Biotechnology (RCB), Faridabad 121001, Delhi-NCR, India; suman.saurav@rcb.res.in (S.S.); bhartijaya00@gmail.com (J.B.S.); 4 Unilever R and D, Bangalore 560066, India; maitreyee.dutta@unilever.com (M.D.); umasan2014@gmail.com (U.S.); joshi.manoj@unilever.com (M.J.); stephen.madison@quinnipiac.edu (S.M.); nirmala.nair@unilever.com (N.N.)

**Keywords:** MFN2, reactive oxygen species, melanogenesis, melanosome, mitochondria

## Abstract

Inter-organellar communication is emerging as one of the most crucial regulators of cellular physiology. One of the key regulators of inter-organellar communication is Mitofusin-2 (MFN2). MFN2 is also involved in mediating mitochondrial fusion–fission dynamics. Further, it facilitates mitochondrial crosstalk with the endoplasmic reticulum, lysosomes and melanosomes, which are lysosome-related organelles specialized in melanin synthesis within melanocytes. However, the role of MFN2 in regulating melanocyte-specific cellular function, i.e., melanogenesis, remains poorly understood. Here, using a B16 mouse melanoma cell line and primary human melanocytes, we report that MFN2 negatively regulates melanogenesis. Both the transient and stable knockdown of MFN2 leads to enhanced melanogenesis, which is associated with an increase in the number of mature (stage III and IV) melanosomes and the augmented expression of key melanogenic enzymes. Further, the ectopic expression of MFN2 in MFN2-silenced cells leads to the complete rescue of the phenotype at the cellular and molecular levels. Mechanistically, MFN2-silencing elevates mitochondrial reactive-oxygen-species (ROS) levels which in turn increases melanogenesis. ROS quenching with the antioxidant N-acetyl cysteine (NAC) reverses the MFN2-knockdown-mediated increase in melanogenesis. Moreover, MFN2 expression is significantly lower in the darkly pigmented primary human melanocytes in comparison to lightly pigmented melanocytes, highlighting a potential contribution of lower MFN2 levels to higher physiological pigmentation. Taken together, our work establishes MFN2 as a novel negative regulator of melanogenesis.

## 1. Introduction

Cells maintain their integrity and function by coordinating various metabolic, chemical and cellular pathways. Molecular crosstalk between different organelles enables cells to process and interpret multiple biological inputs differently in order to modulate cellular physiology [1]. Different biological cues influence cellular properties by rewiring the inter-organellar communication required for cellular homeostasis and function [1,2]. One of the key players involved in the crosstalk between several organelles is MFN2. It can regulate inter-organellar communication between mitochondria and various organelles such as the endoplasmic reticulum and lysosomes [3,4,5,6]. Moreover, MFN2 is a dynamin-related GTPase that resides on the outer mitochondrial membrane, where it plays a critical role in the fusion of mitochondria [7]. 

Recently, Daniele et al. reported that MFN2 is an essential component of mitochondria–melanosome interactions [8]. Melanosomes are specialized lysosome-related organelles present in melanocytes and are the site of melanogenesis (melanin synthesis) [9]. Melanogenesis is a highly conserved process that provides protection from UV radiation [10,11]. The process of melanin synthesis is a free-radical-based mechanism and melanin itself can scavenge free-radicals as well. Further, reactive-oxygen-species (ROS) generation can potentiate melanogenesis [12,13]. Melanin, the end product of complex multistep transformations of L-tyrosine, is synthesized by melanosomes through the hydroxylation of tyrosine or the oxidation of 3,4-dihydroxyphenilalanine (DOPA) by the tyrosinase enzyme [14,15]. Melanosome biogenesis is marked by four characteristic stages (stages I–IV) that exhibit a progressive increase in melanin content [16]. This process of melanosome maturation is coupled with the distinct intracellular distribution of the organelle. The immature, minimally pigmented melanosomes (stage I and II) are predominately present in the cell center while mature, heavily pigmented melanosomes (stage III and IV) are majorly localized at the distal ends of the melanocyte dendrites. The mature melanosomes are then transported to nearby keratinocytes in order to provide protection from UV radiation.

It has been reported that mitochondria–melanosome interactions are more prominent in the case of immature melanosomes (Stage I and II) in comparison to mature melanosomes (Stage III and IV) [8]. Further, mitochondria–melanosome interactions are tethered by MFN2, as MFN2 silencing drastically reduces this inter-organellar bridging [8]. Taken together, the literature suggests that MFN2 plays a critical role in mitochondria–melanosome tethering. However, the functional significance of MFN2 in melanocyte cell biology remains poorly understood. Although Daniele et al. reported that MFN2 positively regulates melanosome biogenesis, they did not specify MFN2’s role in controlling melanosome maturation and the overall melanogenesis process [8]. Moreover, the authors performed MFN2-knockdown studies in mouse melanocytes with the Ocular Albinism 1 (OA1, G-protein-coupled receptor with an established role in ocular albinism) knockout background. Their conclusions were drawn on the basis of MFN2-silencing studies performed under OA1-rescue conditions in OA1-knockout mouse cells. Importantly, the authors did not perform any MFN2 experiments in either wild-type melanocytes or primary human melanocytes [8]. Therefore, the aim of this study was to investigate the role of MFN2 in melanogenesis using different melanogenesis models including primary human melanocytes. 

## 2. Materials and Methods

### 2.1. Cell Culture

B16-F10 cells were obtained from ATCC and cultured in DMEM (Sigma, Bangalore, India). Trypsin, Dulbecco’s Phosphate Buffer Saline (DPBS), Versene, Fetal Bovine Serum (FBS) and additional cell culture grade reagents were obtained from Invitrogen, Waltham, MA, USA. Cells were cultured in DMEM medium supplemented with 10% Fetal Bovine Serum (heat inactivated) at 60–80% confluence and at 5% CO_2_ levels. To set up the Low-Density (LD) pigmentation-oscillator model of B16s, cells were seeded at 100 cells/cm^2^ in DMEM supplemented with 10% Fetal Bovine Serum as described earlier [17,18]. 

Lightly and darkly pigmenting neonatal primary human melanocytes (LP and DP respectively) were procured from Invitrogen, Waltham, MA, USA. Cells were grown in Medium 254 supplemented with human melanocyte growth supplement-2 and maintained at 37 °C in a humidified incubator with 5% CO_2_ atmosphere. The maintenance and sub culturing of cells were carried out as per the manufacturer’s instructions. Cells between passages 3–6 were used for experimentation. 

### 2.2. siRNA-Based Transient Transfections

siRNA transfections were performed in B16 cells and primary human melanocytes using smartpool siRNAs from Dharmacon. Dharmafect was used for transfecting B16 cells and primary human melanocytes. siRNA and transfection reagent where mixed and incubated over the cells in OptiMEM (Gibco, Waltham, MA, USA) media for 4–6 h for achieving optimal transfection efficiency. The siRNAs (smartpool of 4 individual siRNAs targeting gene of interest) were procured from Dharmacon. The catalog number of siRNAs used in the study are included in Table 1.

### 2.3. Lentiviral Based Stable Cell Line Generation

Lentiviral packaging and transduction were performed as previously reported [19,20,21]. Briefly, lentiviral packaging was carried out by co-transfecting pVSVG, pdR8.2 and the desired shRNA clone in HEK cells. Two days post transfections; lentiviral particles were collected from the cell supernatant and were used for transducing the B16 cell line. Finally, stable cell lines were generated by puromycin-based antibiotic selection. Western blots were performed for validating stable-cell-line generation and knockdown efficiency. The GIPZ lentiviral shRNAs were commercially procured from Dharmacon, Lafayette, CO, USA. The shRNA sequences and their catalog numbers are provided in Table 2.

### 2.4. MFN2 Rescue Experimentation

Human YFP tagged MFN2 plasmid (2 μg) was overexpressed in stable shMFN2 B16 cells on day 3 of LD-pigmentation model. The effect of MFN2 rescue was analyzed on LD day 6 by performing melanin-content assays and western blotting for key melanogenic enzymes. Human Mfn2-YFP was a gift from Richard Youle (Addgene plasmid # 28010). 

### 2.5. Western Blotting

Cells were lysed using NP40 lysis buffer supplemented with protease inhibitors. Typically, 50–100 μg proteins were subjected to SDS-PAGE (7.5–10%). Proteins from gels were then electro-transferred onto PVDF membranes. After blocking with 5% non-fat dry milk (NFDM) dissolved in Tris-buffered saline containing 0.1% Tween 20 (TTBS), blots were probed overnight at 4 °C, with specific primary antibodies in TTBS containing 2% NFDM. The primary antibodies used were typically procured from Abcam and were used at 1:500–1:2000 dilutions. The following day, membranes were incubated for 2 h at room temperature with a horseradish-peroxidase-conjugated anti-mouse or anti-rabbit IgG antibody in TTBS containing 2% NFDM. Detection was performed using the enhanced chemiluminescence reagent (ECL Western blotting detection reagents; Amersham Biosciences). Quantification of bands was performed by densitometry using the ImageJ software. The catalogue number and company name for the antibodies are provided in Table 3.

### 2.6. Estimation of ROS

B16 cells were grown in the LD experimental setup (100 cells/cm^2^) in T75 flasks filled with the appropriate culture medium. Either shNT control or stable shMFN2 B16 cells were seeded for LD experiments. LD-day-0 cell pellets were also prepared for basal readout of mitochondrial ROS. Mitochondrial ROS were detected on LD day 0, day 4 and day 7 in these cells using MitoSOX™ (#M36008; Molecular probes, ThermoScientific). The MitoSOX™ staining was performed according to manufacturer’s instructions. Briefly, cells were incubated for 20 min at 37 °C in the dark. Next, cells were washed gently three times with warm buffer. The cells were then trypsinized using standard trypsinization protocol for B16 cells, washed with 1X PBS, resuspended in the appropriate amount of 1X PBS and analyzed using flow cytometry. 

### 2.7. Tyrosinase Activity/DOPA Assay

Tyrosinase-enzyme activity was checked in cell lysates by performing DOPA assay as previously reported [22]. Briefly, cell lysates were prepared in NP-40 lysis buffer and an equal amount of protein was run on a gel under nonreducing/native conditions. The gel was then immersed in phosphate buffer supplemented with tyrosinase substrate L-DOPA (Sigma Chemicals, Bangalore, India). Enzyme activity corresponded to the formation of black-color pigment.

### 2.8. Melanin-Content Assay

Melanin-content assay was performed as described earlier [23]. The cells were lysed in 1N NaOH by heating at 80 °C for 2 hours and then absorbance was measured at 405 nm. Melanin content was estimated by interpolating the sample readings on the melanin standard curve (μg/mL) obtained with synthetic melanin. 

### 2.9. N-Acetyl Cysteine (NAC) Treatment for Scavenging ROS

Stable B16 cell lines were used for setting up the LD melanogenesis assay. NAC (Sigma, A7250) treatment was given on LD days 1, 3 and 5 at 2 mM concentration, dissolved in autoclaved Milli Q water, pH 7.4–7.6. These experiments were terminated on LD day 7 followed by cell count and acquisition of pellet pictures. To measure changes in the mitochondrial ROS levels upon NAC treatment, MitoSOX™ dye was added to cells terminated on LD Day 4 and ROS levels were estimated as described in the earlier section.

### 2.10. Transmission Electron Microscopy

Transmission electron microscopy was performed on shControl and stable shMFN2 B16 cells on LD day 7 using standard protocols. Briefly, cells were fixed overnight in fixative containing 2.5% glutaraldehyde and 4% paraformaldehyde, gradually dehydrated in graded series of ethanol and embedded in Epon 812 resin. Ultrathin sections were cut and stained with uranyl acetate and lead citrate and images were captured using a transmission electron microscope (Tecnai twin 20, FEI). 

### 2.11. Confocal Microscopy 

For examination of the mitochondrial morphology of B16 shNT control cells, shMFN2 and stable shDRP1 cells were cultured in Lab-Tek 2-chambered coverglass (Thermo Scientific, Waltham, MA, USA) plates. Cells were then stained with 0.1 μM/100 nM MitoTracker^TM^ Red FM (Invitrogen, Waltham, MA, USA) for 30 min at 37 °C in complete culture medium, protected from light. Further, cells were gently washed with 1X HBSS three times and imaged on a Zeiss LSN 880 confocal microscope using 63X oil objective.

### 2.12. Statistical Analysis

All experiments were performed at least 3 times. Data are presented as mean ± SEM and the unpaired student’s *t*-test was performed for determining statistical significance between 2 experimental samples, whereas one-way ANOVA was performed for the comparison of 3 samples. A *p*-value < 0.05 was considered as significant and is presented as “*”; *p*-value < 0.01 is presented as “**”and *p*-value < 0.001 is presented as “***”. 

## 3. Results

### 3.1. MFN2 Expression Is Inversely Related to Pigmentation Levels

**Figure 1:**
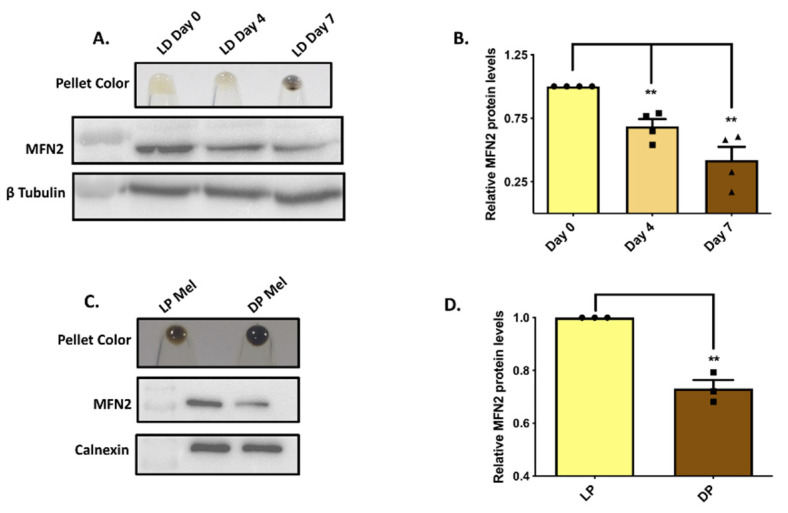
**MFN2 expression is inversely associated with pigmentation.** (**A**). Representative pellet pictures of B16 cells on different days of LD pigmentation and MFN2-protein expression in the LD-pigmentation model (N = 4). (**B**) Densitometry quantitation of MFN2 levels during LD pigmentation (N = 4). (**C**) Representative pellet pictures of lightly pigmented (LP) and darkly pigmented (DP) primary human melanocytes and MFN2-protein expression in the LP-DP primary human melanocytes (N = 3). (**D**) Densitometry quantitation of MFN2 levels in LP-DP primary human melanocytes (N = 3). Data presented are mean ± S.E.M. (** *p* < 0.01; unpaired student’s *t*-test was performed for determining statistical significance between two experimental samples, whereas one-way ANOVA was performed for comparison of three samples).

We have recently established a B16-mouse-melanoma-cell-line-based low-density (LD)-culturing-induced-pigmentation model [17]. This model closely recapitulates the melanogenic pathways and signaling cascades operating in primary human melanocytes [17,18]. The LD-pigmentation model leads to a gradual increase in pigmentation over a seven-day period wherein the day-zero (D0) cells are depigmented by day four (D4), and the pigmentation machinery becomes activated and the cells are completely pigmented by day seven (D7) (Figure 1A). To evaluate the association of MFN2 expression with pigmentation levels, we examined MFN2-protein levels in the LD-pigmentation model. We observed that as B16 cells became pigmented in the LD model, the expression of MFN2 decreased significantly (Figure 1A,B). This suggests that MFN2 expression is inversely related to pigmentation levels in B16 cells. To evaluate whether this phenomenon is cell-type or species specific, we compared MFN2-protein expression between darkly and lightly pigmented primary human melanocytes. Our data suggest that MFN2 levels are significantly lower in the darkly pigmented primary human melanocytes as compared to lightly pigmented primary human melanocytes (Figure 1C,D). Taken together, these findings clearly demonstrate an inverse relationship between MFN2 expression and pigmentation levels. 

### 3.2. MFN2 Negatively Regulates Pigmentation

**Figure 2:**
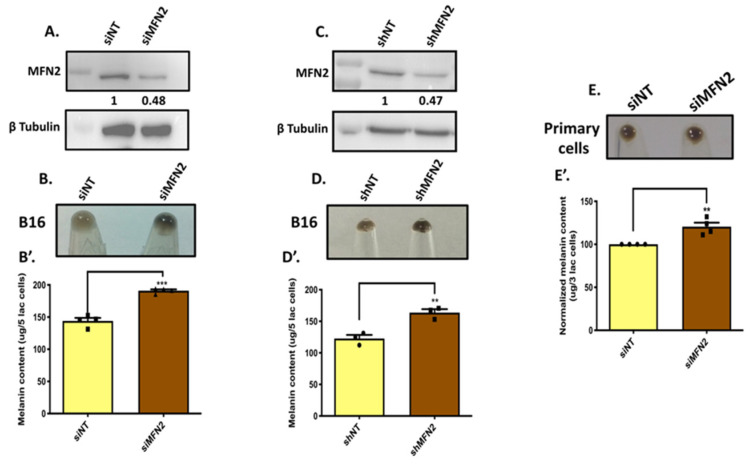
**MFN2 negatively regulates pigmentation.** (**A**) Representative western blot confirming siRNA-based silencing of MFN2 in B16 cells (N = 4). The extent of silencing is quantitated by performing densitometric analysis using ImageJ and is presented below the blot. (**B**) Pellet pictures of siNon-Targeting (siNT) control and siMFN2 on LD day seven (N = 4). (**B’**) Melanin-content estimation of siNT and siMFN2 B16 cells on LD day seven (N = 4). (**C**) Representative western blot validating lentiviral-mediated, shRNA-based stable knockdown of MFN2 in B16 cells (N = 3). The knockdown is quantitated by performing densitometric analysis and is presented below the blot. (**D**) Pellet pictures of shNon-Targeting (shNT) control and shMFN2 on LD day seven (N = 3). (**D’**) Melanin-content estimation of shNT and shMFN2 B16 cells on LD day seven (N = 3). (**E**) Pellet pictures of lightly pigmented primary human melanocytes transfected with either siNT or siMFN2 (N = 4). (**E’**) Melanin-content estimation of lightly pigmented primary human melanocytes transfected with either siNT or siMFN2 (N = 4). Data presented are mean ± S.E.M. (*** *p* < 0.001; ** *p* < 0.01; unpaired student’s *t*-test was performed for statistical analysis).

To examine the role of MFN2 in melanogenesis, we transiently silenced MFN2 using siRNAs. We started by silencing MFN2 using specific siRNA against it in the LD-pigmentation model. We observed a significant decrease in the MFN2 levels with the siRNA transfection in B16 cells (Figure 2A). The knockdown of MFN2 resulted in the visible increase in LD-day-seven melanogenesis as evident in the day-seven pellet pictures (Figure 2B). We further quantitated the increase in melanogenesis upon MFN2 silencing by performing melanin-content assays. We observed that transient MFN2 silencing enhanced melanogenesis by ~35% in B16 cells (Figure 2B’). To further corroborate this observation, we generated lentiviral-based stable B16 cell lines with MFN2 knockdown. We observed a considerable decrease in the MFN2 expression in the stable shMFN2 B16 cells (Figure 2C). We next performed LD-pigmentation assays with either shNon-Targeting (shNT)-expressing stable control cells or stable shMFN2 cells. As expected, stable shMFN2 cells acquired higher melanin levels as observed in the LD-day-seven pellet pictures (Figure 2D). Next, we quantitated the increase in melanogenesis by performing melanin-content assays. We found that the melanin content was increased by ~33% in stable shMFN2 cells in comparison to shNT cells (Figure 2D’). We further validated the negative role of MFN2 in regulating melanogenesis by silencing MFN2 in primary human melanocytes. Using siRNAs targeting human MFN2, we silenced it in both darkly pigmented and lightly pigmented primary human melanocytes. We observed that MFN2 silencing in lightly pigmented primary human melanocytes led to a significant increase in melanogenesis (Figure 2E,E’). However, a similar exercise in darkly pigmented primary human melanocytes did not alter their melanin content. This could be due to the fact that darkly pigmented primary human melanocytes are already highly pigmented, with little or no further scope for increase in pigmentation. Alternately, the endogenous levels of MFN2 in darkly pigmented primary human melanocytes are already too low and therefore any further decrease in its expression does not contribute to any additional pigment synthesis. 

**Figure 3:**
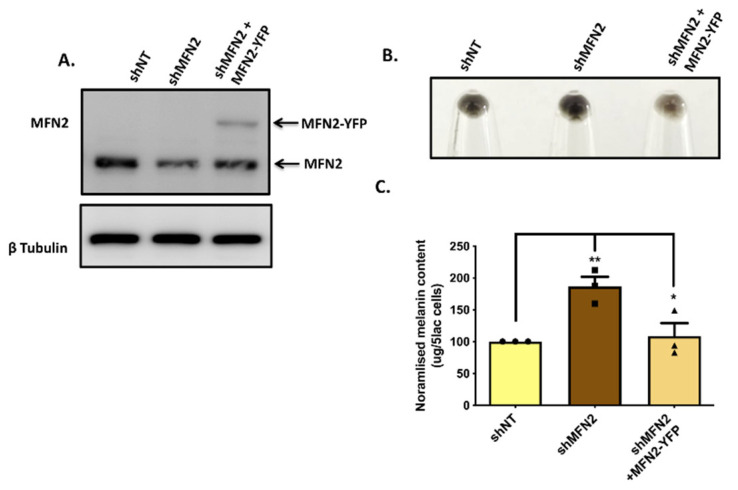
**MFN2 rescue reverses pigmentation phenotype.** (**A**) Representative western blot demonstrating ectopic expression of YFP-tagged human MFN2 in stable shMFN2 B16 cells. (**B**) Pellet pictures of shNT, shMFN2 and shMFN2 plus MFN2–YFP cells on LD day six (N = 3). (**C**) Melanin-content estimation of shNT, shMFN2 and shMFN2 plus MFN2–YFP B16 cells on LD day six (N = 3). Data presented are mean ± S.E.M. (* *p* < 0.05; ** *p* < 0.01; one-way ANOVA was performed was performed for statistical analysis).

Next, we performed MFN2-rescue experiments in order to corroborate the role of MFN2 in melanogenesis. We used the human MFN2–YFP plasmid (Addgene plasmid #28010) for overexpressing MFN2 in stable shMFN2 B16 cells for evaluating the effect of MFN2 rescue on pigmentation in MFN2-silenced cells. We transfected the MFN2 plasmid in stable shMFN2 B16 cells on day three of the LD-pigmentation model. We analyzed the effect of MFN2 rescue on LD day six by performing melanin-content assays and western blotting for key melanogenic enzymes. Firstly, we confirmed MFN2 overexpression in stable shMFN2 B16 cells by performing western blotting. Since MFN2 was tagged with YFP in the overexpression construct, we could observe a higher molecular-weight band corresponding to MFN2–YFP in the rescue condition (Figure 3A), thereby validating MFN2 rescue in the shMFN2 cells. Next, we examined the effect of MFN2 rescue on the LD pigmentation. Excitingly, we could almost completely reverse the effect of MFN2 knockdown upon the ectopic expression of MFN2 in these cells. The pellet pictures from shNT, shMFN2 and shMFN2+MFN2–YFP conditions clearly showed rescue of the phenotype in shMFN2+MFN2–YFP cells (Figure 3B). We further quantitated these phenotypic changes by performing melanin-content assays and observed that indeed, the ectopic expression of MFN2–YFP in shMFN2 cells led to a significant decrease in melanogenesis (Figure 3C). These data further substantiate that MFN2 is a negative regulator of pigmentation. Taken together, these experiments strongly pointed to a negative role of MFN2 in the melanogenesis process.

### 3.3. MFN2 Silencing Enhances Melanosome Maturation and Melanogenic Enzyme Expression

**Figure 4:**
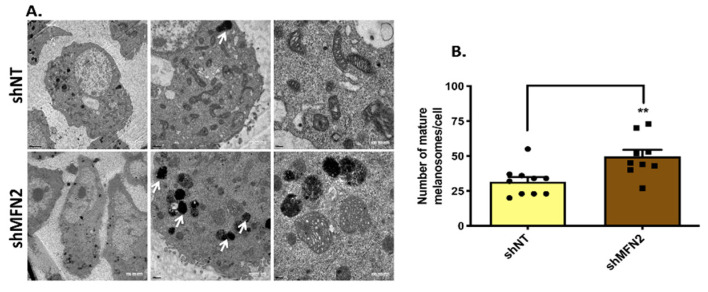
**MFN2 controls melanosome maturation.** (**A**) TEM images of shNT and stable shMFN2 B16 cells. White arrows correspond to melanin-rich mature (stage three and four) melanosomes in these cells on LD day seven. (**B**) Quantification of number of mature melanosomes/cells in shNT and stable shMFN2 cells on LD day seven (N = 9 cells). Data presented are mean ± S.E.M. (** *p* < 0.01; unpaired student’s *t*-test was performed for statistical analysis).

In order to decipher the cellular signatures underlying the enhanced melanogenesis upon decrease in MFN2 expression, we evaluated the role of MFN2 in regulating melanosome biogenesis and the expression of key melanogenic enzymes. Transmission-electron-microscopy (TEM)-based ultrastructural studies were performed to evaluate the role of MFN2 in melanosome biogenesis and maturation. Specifically, the ultrastructural examination of control shNT and stable shMFN2 B16 cells was performed on LD day seven and stage-three and stage-four melanosomes (electron dense) were counted manually. It was observed that MFN2 knockdown led to an increase in the number of mature (stage three and four) melanosomes (Figure 4A,B), suggesting that MFN2 negatively regulates melanosome maturation. Interestingly, in an earlier study, MFN2-mediated mitochondria–melanosome interactions were reported between mitochondria and stage I and II melanosomes [8]. It was further demonstrated that stage III and IV melanosomes rarely associate with mitochondria. Based on our data, it could be possible that the lack of physical interaction between mature melanosomes and mitochondria is coupled with a decrease in MFN2 levels. However, further studies in distinct model systems will be required in order to confirm this hypothesis. 

**Figure 5:**
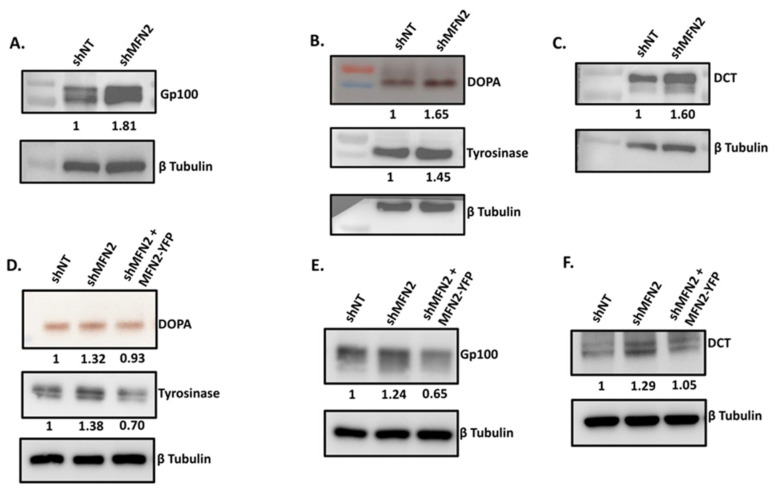
**MFN2 regulates melanogenic enzyme expression.** (**A**) Representative western blot showing expression of Gp100 shNT and stable shMFN2 cells on LD day seven (N = 3). (**B**) DOPA assay showing activity of tyrosinase enzyme and western blot for tyrosinase expression in shNT and stable shMFN2 cells on LD day seven (N = 3). (**C**) Representative western blot showing expression of DCT in shNT and stable shMFN2 cells on LD day seven (N = 3). (**D**) DOPA assay showing activity of tyrosinase enzyme and western blot for tyrosinase expression in shNT, shMFN2 and shMFN2 plus MFN2–YFP cells on LD day six (N = 3). (**E**) Representative western blot showing expression of Gp100 in shNT, shMFN2 and shMFN2 plus MFN2–YFP cells on LD day six (N = 3). (**F**) Representative western blot showing expression of DCT in shNT, shMFN2 and shMFN2 plus MFN2–YFP cells on LD day six (N = 3). The activity and expression of the key melanogenic proteins was quantitated by performing densitometric analysis using ImageJ and is presented below the gels/blots.

We next examined the expression of key melanosomal structural proteins as well as melanogenic enzymes upon the silencing of MFN2. We performed western-blot analysis in order to evaluate the levels of Premelanosome Protein 17 (PMEL17 or Gp100), which is a melanocyte-specific structural glycoprotein that is essential for melanosome biogenesis, maturation and formation of melanosome striations [24]. As presented in Figure 5A, stable shMFN2 cells expressed significantly higher levels of Gp100 as compared to stable control shNT on LD day seven. These data corroborated the TEM analysis wherein MFN2 knockdown led to higher melanosome biogenesis and maturation. Further, we evaluated the expression and activity of the rate-limiting enzyme in the melanin-synthesis pathway, i.e., tyrosinase [25]. We performed a DOPA (dopachrome) assay to examine tyrosinase activity in shNT control and stable shMFN2 cells on LD day seven. Tyrosinase converts substrate L-DOPA to Dopachrome that yields a brown-to-black color on native gels. We observed that both the expression and activity of tyrosinase was significantly increased in shMFN2 cells in comparison to shNT control cells (Figure 5B). Finally, we analyzed the expression of melanogenic enzyme tyrosinase-related protein 2/Dopachrome Tautomerase (DCT) in the shNT and stable shMFN2 cells. We observed that MFN2 silencing results in the significant enhancement in the expression of DCT (Figure 5C). The quantitation of the extent of the increase in key melanogenic proteins upon MFN2 knockdown was performed on several independent blots. These quantitative data are presented in Supplementary Figure S1. To further corroborate the role of MFN2 in regulating the expression of key melanogenic enzymes, we carried out their expression analysis under MFN2-rescue conditions. To investigate tyrosinase activity and expression, we performed DOPA assay and tyrosinase protein analysis, respectively. As expected, we observed a substantial decrease in tyrosinase activity and expression upon the ectopic expression of MFN2 in stable shMFN2 cells (Figure 5D). Next, we examined the levels of Gp100 and DCT upon MFN2 rescue. In these experiments, we saw a significant decrease in Gp100 and DCT-protein expression under MFN2-rescue conditions when compared to stable shMFN2 cells (Figure 5E,F). Please refer to supplementary data for the raw/full blot images of all the western-blotting data presented in the manuscript. Taken together, these findings suggested that the increase in both melanosome maturation and expression of key melanogenic enzymes contribute to enhanced melanogenesis in the MFN2-knockdown cells. Moreover, these studies further strengthened the negative role of MFN2 in pigmentation.

### 3.4. MFN2 Regulates Melanogenesis Independent of Mitochondrial Dynamics

**Figure 6:**
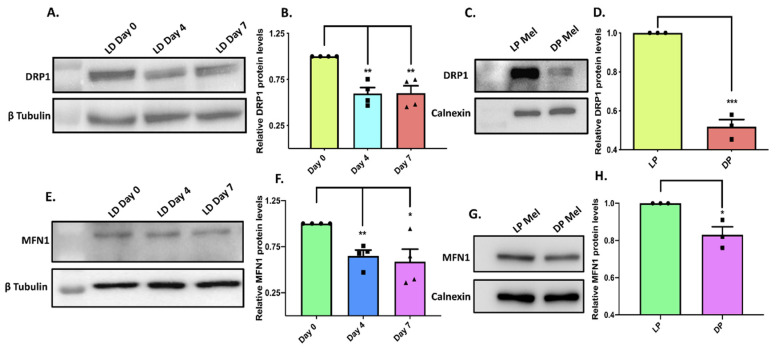
**DRP1 and MFN1 expression decreases with pigmentation.** (**A**) Representative western blot showing DRP1-protein expression in the LD-pigmentation model (N = 4). (**B**) Densitometric quantitation of DRP1 levels during LD pigmentation (N = 4). (**C**) Representative blot showing DRP1-protein expression in the LP-DP primary human melanocytes (N = 3). (**D**) Densitometric quantitation of DRP1 levels in LP-DP primary human melanocytes (N = 3). (**E**) Representative western blot showing MFN1 protein expression in the LD-pigmentation model (N = 4). (**F**) Densitometric quantitation of MFN1 levels during LD pigmentation (N = 4). (**G**) Representative blot showing MFN1 protein expression in the LP-DP primary human melanocytes (N = 3). (**H**) Densitometric quantitation of DRP1 levels in LP-DP primary human melanocytes (N = 3). Data presented are mean ± S.E.M. (* *p* < 0.05; ** *p* < 0.01; *** *p* < 0.001 unpaired student’s *t*-test was performed for statistical analysis).

Next, we directed our efforts to identify the molecular choreography leading to enhanced melanogenesis upon MFN2 silencing. Since the most well-studied molecular function of MFN2 is to regulate mitochondrial dynamics (mitochondrial fusion and fission), we evaluated the role of mitochondrial dynamics in modulating melanogenesis in our model system. MFN2 is essential for mitochondrial fusion and dynamin-related protein 1(DRP1) plays a critical role in mitochondrial fission [26]. Likewise, Mitofusin1 (MFN1) is a homolog of MFN2 that plays an important role in mitochondrial fusion [26]. Therefore, to understand the role of mitochondrial dynamics, we decided to study the functional significance of DRP1 and MFN1 in melanogenesis. If MFN2′s negative role in pigmentation is associated with its crucial role in mitochondrial dynamics, then DRP1 and MFN1 should also be involved in regulating melanogenesis. First of all, we examined the expression profile of DRP1 expression in the B16 LD-pigmentation model. Interestingly, DRP1-protein levels decreased with the increase in melanogenesis, similar to that observed in the case of MFN2 (Figure 6A,B). We next evaluated DRP1 expression in darkly and lightly pigmented primary human melanocytes. We again observed that similar to MFN2, DRP1-protein levels were lower in darkly pigmented as compared to lightly pigmented primary human melanocytes (Figure 6C,D). We performed a similar expression analysis of MFN1 in the LD-pigmentation model and in differentially pigmented primary human melanocytes. We observed that the expression of MFN1 decreased with the increase in pigmentation in both the LD-pigmentation model (Figure 6E,F) and darkly pigmented primary human melanocytes (Figure 6G,H). These experiments suggest that the expression profiles of MFN1 and DRP1 follow the same pattern as MFN2 in melanocytes of differential pigmentation status. 

**Figure 7:**
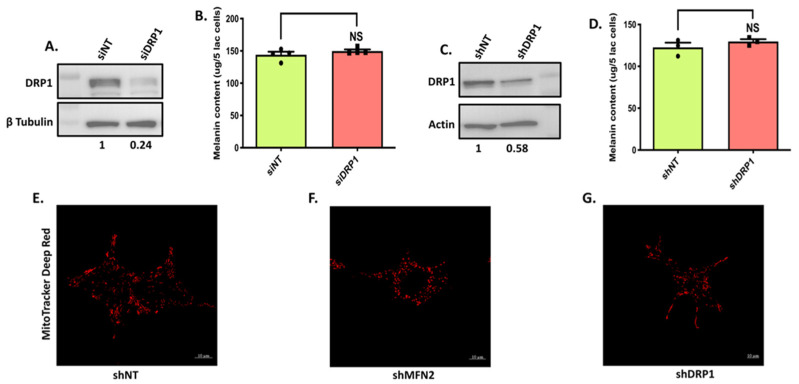
**DRP1 does not regulate melanogenesis.** (**A**) Representative western blot confirming siRNA-based silencing of DRP1 in B16 cells (N = 4). The extent of silencing is quantitated by performing densitometric analysis using ImageJ and is presented below the blot. (**B**) Melanin-content estimation of siNT and siDRP1 B16 cells on LD day seven (N = 4). (**C**) Representative western blot validating lentiviral-mediated shRNA-based stable knockdown of DRP1 in B16 cells (N = 3). The extent of DRP1 knockdown is quantitated by performing densitometric analysis and is presented below the blot. (**D**) Melanin-content estimation of shNT and shDRP1 B16 cells on LD day seven (N = 3). (**E**–**G**) Representative confocal-microscopy images of shNT, shMFN2 and stable shDRP1 cells stained with MitoTracker Deep Red for examining mitochondrial shape (N = 20–25 cells/condition). Data presented are mean ± S.E.M. (NS means Not significant; unpaired student’s *t*-test was performed for statistical analysis).

To further understand the role of mitochondrial dynamics in melanogenesis, we performed transient DRP1 silencing with siRNA specifically targeting DRP1 in the LD-pigmentation model (Figure 7A). As presented in Figure 7B, DRP1 knockdown did not significantly affect melanogenesis. We next generated lentiviral-based shDRP1 B16 cell lines for the stable silencing of DRP1 and used these cells for evaluating DRP1′s role in pigmentation. We first confirmed the knockdown of DRP1 in the stable shDRP1 cells (Figure 7C). We then compared the LD-day-seven melanogenesis levels between shNT control cells and stable shDRP1 cells by performing melanin-content assay (Figure 7D). Interestingly, even a stable knockdown of DRP1 did not alter melanogenesis in our model system. This suggests that MFN2 is most likely regulating melanogenesis independent of its established role in mitochondrial dynamics. However, one distant possibility could be that MFN2 and DRP1 were not playing their classical roles in mitochondrial dynamics in the B16 model. Therefore, we evaluated the status of mitochondrial dynamics in shMFN2 and stable shDRP1 B16 cells by performing confocal microscopy. We stained shNT control, shMFN2 and stable shDRP1 cells with MitoTracker Deep Red for examining the mitochondrial structure in these cells. As reported in several systems, shMFN2 cells (Figure 7F) showed fragmented mitochondria whereas shDRP1 cells displayed elongated mitochondria (Figure 7G) as compared to shNT control cells (Figure 7E). These data validated the opposing roles played by MFN2 and DRP1 vis a vis the modulation of mitochondrial dynamics and highlighted the validity of the B16 model system to probe the molecular mechanisms underlying MFN2-induced melanogenesis. On the other hand, the observed lack of antagonism in the regulation of melanogenesis by MFN2 and DRP1 strengthened the case for a mitochondrial-dynamics-independent mechanism for the regulation of melanogenesis by MFN2. We therefore started exploring alternate mechanisms that could explain hyper-melanogenesis associated with the decrease in MFN2 expression. 

### 3.5. MNF2 Regulates Melanogenesis by Modulating Mitochondrial ROS 

Apart from mitochondrial dynamics, MFN2 has been reported to regulate mitochondrial reactive-oxygen-species (ROS) generation and thereby overall cellular ROS levels [2,27]. Melanogenesis is an energy-intensive process that is expected to increase mitochondrial activity. However, while mitochondria power the oxidative phosphorylation, this energetic process is somewhat “leaky,” and the electron-transport chain generates free radicals that contribute to an overall increase in reactive species within both the mitochondrial matrix and cytosol [28]. Elevated ROS, in turn, have been implicated in promoting melanogenesis as in the case of the stimulation of melanogenesis by UVA [12]. Interestingly, ROS levels are known to modulate melanogenesis by regulating the MITF-mediated transcriptional up-regulation of melanogenic proteins and by enhancing the expression as well as the activity of tyrosinase [12,13]. As we observed an increase in tyrosinase activity and expression upon MFN2 silencing, we asked the question if MFN2-knockdown-mediated hyper-melanogenesis is associated with changes in ROS levels. 

**Figure 8:**
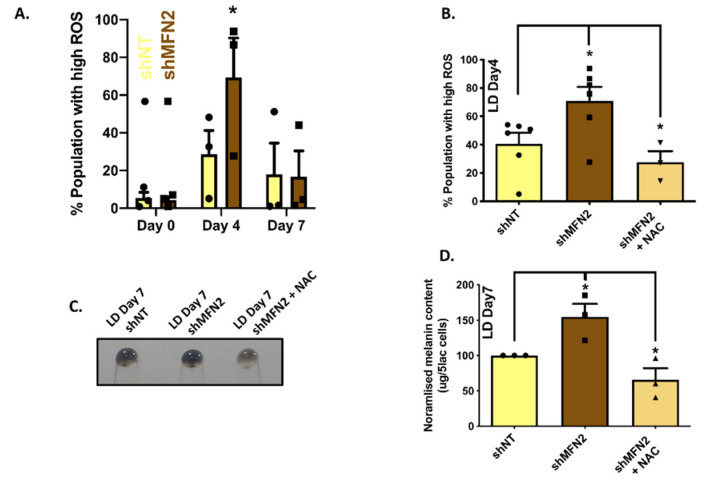
**MFN2 regulates melanogenesis by modulating ROS levels.** (**A**) Estimation of ROS levels in shNT and shMFN2 cells during LD-pigmentation model (N = 3). (**B**) ROS estimation in shNT- (N = 6), shMFN2- (N = 6) and shMFN2+NAC-treated cells (N = 3) on LD day four. (**C**) Pellet pictures of shNT-, shMFN2- and shMFN2+NAC-treated cells on LD day seven (N = 3). (**D**) Melanin-content estimation of shNT-, shMFN2- and shMFN2+NAC-treated cells on LD day seven (N = 3). Data presented are mean ± S.E.M. (* *p* < 0.05; one-way ANOVA was performed was performed for statistical analysis).

We measured mitochondrial ROS levels using MitoSOX, a mitochondrial ROS-measuring dye. We performed flow cytometry on control shNT and stable shMFN2 cells while they were undergoing melanogenesis in the LD-pigmentation model. The time-course analysis of mitochondrial ROS was performed on LD day zero, day four and day seven. We observed that the ROS levels increased as cells pigmented in the LD model with the highest ROS levels on LD day four (Figure 8A). Interestingly, ROS levels in stable shMFN2 cells were significantly higher in comparison to shNT cells on LD day four, whereas the levels were comparable on day zero and day seven (Figure 8A). This observation raised the possibility that higher ROS levels in the case of stable shMFN2 cells could be contributing to an increase in melanogenesis observed in these cells. Therefore, we performed rescue experiments by scavenging ROS levels during the course of LD pigmentation with antioxidant N-Acetyl Cysteine (NAC). We first standardized NAC treatment in the LD model for quenching ROS and we were able to completely attenuate the shMFN2-induced rise in ROS levels (Figure 8B). We next evaluated the effect of the NAC treatment on MFN2-silencing-stimulated enhanced melanogenesis. Excitingly, NAC treatment drastically reduced the increase in melanogenesis (Figure 8C,D) observed in stable shMFN2 cells. Taken together, these findings indicate that the rise in ROS levels upon MFN2 silencing was at least partially responsible for the enhanced melanogenesis in these cells.

## 4. Discussion

Inter-organellar crosstalk has emerged as a critical regulator of cellular physiology and function. MFN2 is a critical regulator of inter-organelle interactions, in particular mitochondrial communication with other organelles. Recently, Daniele et al. reported that mitochondria and melanosomes physically interact via MFN2-mediated bridges [8]. In this study, we evaluated the functional significance of MFN2 in regulating melanogenesis, a melanocyte-specific cell function. We employed a well-established B16 LD-pigmentation model and primary human melanocytes of two different ethnic origins to explore the role of MFN2 in melanogenesis. Our data show that MFN2 silencing results in enhanced melanogenesis in both B16 cells (Figure 2A–D’) and in primary human melanocytes (Figure 2E,E’). The increase in pigmentation was associated with enhanced melanosome maturation (Figure 4A,B) and elevated levels of key melanogenic enzymes (Figure 5A–C). Although it was previously reported that MFN2 positively regulates melanosome biogenesis, the role of MFN2 in controlling melanosome maturation and the overall melanogenesis process remains largely unknown [8]. Importantly, the earlier conclusions were drawn on the basis of MFN2-silencing studies performed under OA1-rescue conditions in OA1-knockout mouse cells. Therefore, the aberrations that were reported upon MFN2 silencing could be due to the use of mouse melanocytes with the OA1-knockout background and could be a very peculiar phenomenon associated with OA1 knockout. 

Further, it is reported that the MFN2-mediated mitochondria–melanosome interaction is important for ATP transfer during the early stages of melanosome biogenesis [8]. Moreover, the authors showed that mitochondria–melanosome interactions are more important for stage I and II melanosomes and that these interactions are decreased in mature melanosomes (stage III and IV), which could be due to reduction in MFN2 levels. Indeed, the data presented in this study show that MFN2 silencing boosts melanosome maturation (Figure 4). Based on our data, it could be possible that the lack of physical interaction between mature melanosomes and mitochondria is coupled with decrease in MFN2 levels. However, further studies in distinct model systems will be required in order to confirm this hypothesis. 

Our data suggest that MFN2 silencing increases melanogenesis by enhancing number of matured melanosomes (Figure 4A,B) and by augmenting the expression of tyrosinase, Gp100 and DCT (Figure 5A–C). Further, the rescue of MFN2 by its ectopic overexpression in stable shMFN2 cells led to a reversal of the melanogenesis process both at the cellular (Figure 3) and molecular levels (Figure 5D–F). Interestingly, MFN2 knockdown increased cellular ROS levels (Figure 8A) and ROS quenching by NAC completely abrogated MFN2-silencing-induced melanogenesis (Figure 8B–D). This implies that MFN2 modulates cellular oxidative stress, which in turn potentiates melanogenesis. Interestingly, increased ROS levels have been reported to regulate the MITF-mediated expression of melanogenic enzymes including tyrosinase [12,13]. Therefore, the enhanced tyrosinase expression and activity observed upon MFN2 silencing could be due to ROS accumulation in response to MFN2 knockdown. Since MFN2 regulates a number of inter-organelle interactions that in turn can modulate a plethora of signaling cascades, further studies are required in order to completely understand the molecular mechanisms working downstream of MFN2 in melanocytes. In this study, based on literature survey, we focused on evaluating the contribution of ROS in regulating melanogenesis downstream of MFN2. In the future, unbiased transcriptomics and proteomics studies would be required for comprehensively delineating the molecular pathways that connect MFN2 to melanogenesis. Another important question that demands further investigation is how MFN2 silencing leads to accumulation of ROS. One possible explanation could be that the MFN2 mediated mitochondria-melanosome tethering might be playing a role in regulating ROS homeostasis in melanocytes. However, further studies would be required in order to precisely define the role of MFN2 in modulating redox homeostasis in melanocytes. 

An intriguing observation from our studies is that MFN2 might be regulating melanogenesis independently of its established role in mitochondrial dynamics. Dynamin-related protein 1 (DRP1), a member of the dynamin family of GTPases, is the master regulator of mitochondrial fission [29,30]. On the other hand, MFN1/2 regulate outer mitochondrial membrane fusion [31,32]. The expression analysis of these key mitochondrial-dynamics regulators (MFN2, DRP1 and MFN1) during LD pigmentation and in primary human melanocytes suggest that they follow a similar expression profile in differentially pigmented cells (Figure 1 and Figure 6). In general, mitochondrial fission and fusion dynamics regulate cellular functions in opposing manners. However, our data suggests that MFN2 negatively regulates melanogenesis whereas DRP1 does not play a significant role in regulating melanogenesis. In an earlier study, DRP1 was shown to negatively regulate melanogenesis while optic atrophy type 1 (OPA1; facilitates inner mitochondrial membrane fusion) was shown to positively control alpha-melanocyte-stimulating-hormone (α-MSH)-induced melanogenesis [33], suggesting that in the model system used by these authors, mitochondrial dynamics could have been involved in calibrating pigmentation. Certainly, more studies are required in order to completely understand the role of mitochondrial dynamics in melanogenesis. Importantly, the role of MFN2 in pigmentation biology remains largely unappreciated. Here, our data from primary human melanocytes and B16 cells suggest that MFN2 negatively regulates melanogenesis. Moreover, lower MFN2 levels in darkly pigmented primary human melanocytes indicate a physiological role of MFN2 in pigmentation. 

Typically, knockout (KO) mice models are considered as one of the best ways to validate the role of a specific protein in driving a particular cell function. Therefore, we evaluated the published phenotypes associated with MFN2-knockout mice models. Complete-MFN2-KO mice are prenatally lethal, suggesting that MFN2 is critical for embryonic development [7]. However, when we analyzed the phenotypes of conditional/tissue-specific MFN2-KO mice on the Mouse Genome Informatics (MGI) portal, we observed pigmentation as one of the associated phenotypes (http://www.informatics.jax.org/marker/MGI:2442230 Date accessed: 11 June 2021). Using a cardiomyocyte-specific MFN2-KO mice model, Zhao et al. reported that MFN2 ablation leads to the accumulation of brownish pigment in the lysosomes [34]. This brownish pigment was lipofuscin, the aging-related pigment, suggesting that MFN2 could regulate lipofuscin turnover. Recently, Jiang et al. demonstrated that conditional KO of MFN2 in the hippocampus and brain cortex leads to ROS accumulation and oxidative stress in these tissues, thereby emphasizing a critical role of MFN2 in regulating ROS generation [35]. Taken together, these studies corroborate that MFN2 regulates ROS production and pigment accumulation in vivo. In the future, it would be interesting to investigate the significance of MFN2 in pigmentary disorders and aging-associated hyper-pigmentation. 

## 5. Conclusions

We here show that MFN2 negatively regulates melanogenesis. Using primary human melanocytes and B16 cells, we report that either the transient or stable silencing of MFN2 leads to enhanced melanogenesis. This increase in melanogenesis is due to augmented melanosome maturation and the elevated expression of key melanogenic enzymes. Importantly, MFN2 overexpression in MFN2-silenced cells completely rescued the enhanced pigmentation observed upon MFN2 knockdown. The increased melanogenesis upon MFN2 silencing appears to be independent of MFN2′s role in mitochondrial dynamics. Mechanistically, MFN2 knockdown enhances ROS levels and that, in turn, potentiates melanogenesis. Further, ROS scavenging by antioxidant N-Acetyl Cysteine (NAC) completely reversed the MFN2-knockdown-induced increase in melanin synthesis, thereby suggesting that MFN2 regulates melanogenesis at least partially by modulating oxidative stress. Moreover, MFN2 levels were significantly low in darkly pigmented primary human melanocytes in comparison to lightly pigmented primary human melanocytes, underlying a physiological relationship between lower MFN2 expression and higher melanin levels.

## Figures and Tables

**Table 1:** Details of siRNAs used in the study.

siRNA	Catalog Number
siNT	D-001810-10-20
siMFN2 (Mouse)	L-046303-00-0010
siDRP1 (Mouse)	L-054815-01-0010
siMFN2 (Human)	L-012961-00-0005
siDRP1 (Human)	L-012092-00-0005

**Table 2:** Details of shRNAs used in the study.

shRNA	Sequence	Catalog Number
Mouse shMFN2	TGAGTTCGCTGTCCAACCA	RHS5086-EG170731
Mouse shDRP1	TATCTTCTGGTGAAACGTG	RHS5086-EG74006
shNT	NA	RHS4346

**Table 3:** Details of antibodies used in the study.

Antibody	Company	Catalog Number
DCT	Abcam	ab74073
Gp100	Abcam	ab137078
ß-Tubulin	Abcam	ab21058
MFN2	Abcam	ab124773
DRP1	Abcam	ab56788
MFN1	Abcam	ab104274
Calnexin	Abcam	ab22595

## Data Availability

Data supporting reported results are available on request from the corresponding author.

## Supplementary Materials

The following supporting information can be downloaded at: https://www.mdpi.com/article/10.3390/cells11040701/s1, Figure S1. MFN2 regulates melanogenic enzyme expression and activity. (**A**)**.** Quantitation of densitometric analysis of Gp100 expression in shNT and shMFN2 cells on LD Day7 (N = 3). (**B**)**.** Quantitative analysis of tyrosinase activity in shNT and shMFN2 cells on LD Day7 (N = 3). (**C**)**.** Quantitation of densitometric analysis of tyrosinase expression in shNT and shMFN2 cells on LD Day7 (N = 3). (**D**)**.** Densitometric analysis of DCT expression in shNT and shMFN2 cells on LD Day7 (N = 3). Data presented are Mean ± S.E.M. (* *p* < 0.05; ** *p* < 0.01; Unpaired Student’s *t*-test was performed for statistical analysis). Supplementary data: The raw/full blot images of all the west-ern-blotting data presented in the manuscript.
