# Supplementary material for: Mitofusin-2 Negatively Regulates Melanogenesis by Modulating Mitochondrial ROS Generation
Source: Cells. 2022 Feb 16;11(4):701. doi: 10.3390/cells11040701 (PMC8869806; doi:10.3390/cells11040701)

Supplementary data

Figure 1A. MFN2 protein expression in LD pigmentation model

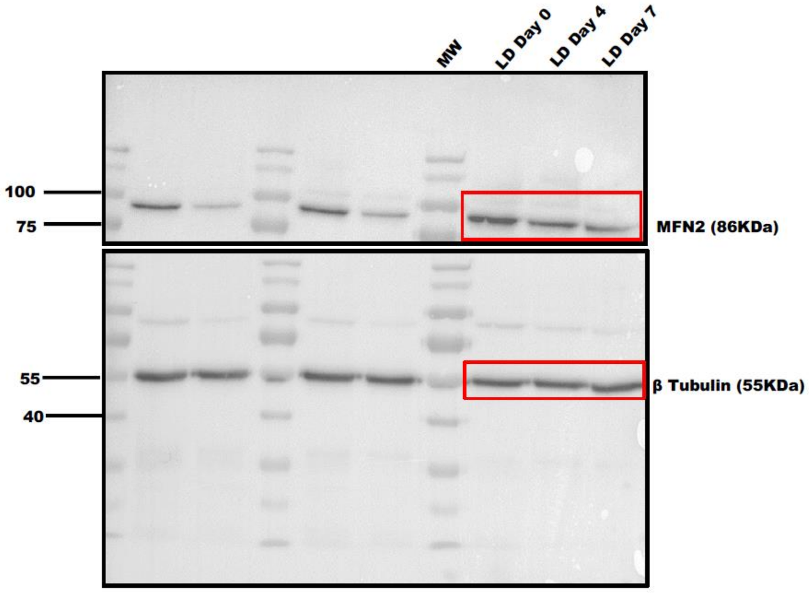

Figure 1C. MFN2 protein expression in LP-DP primary human melanocytes

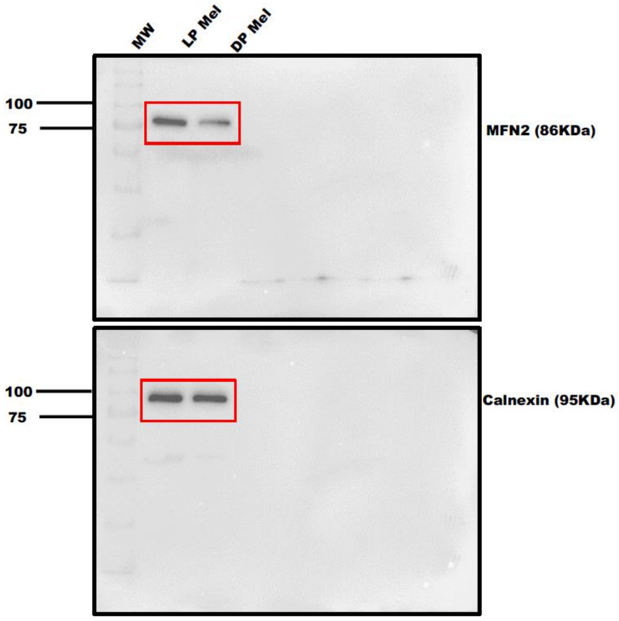

Figure 2A. siRNA based silencing of MFN2 in B16 cells

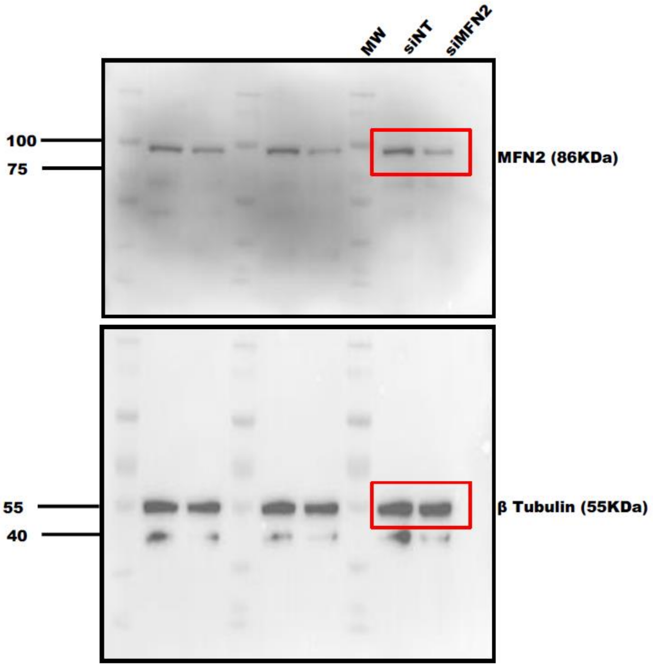

Figure 2C. shRNA mediated stable knockdown of MFN2 in B16 cells

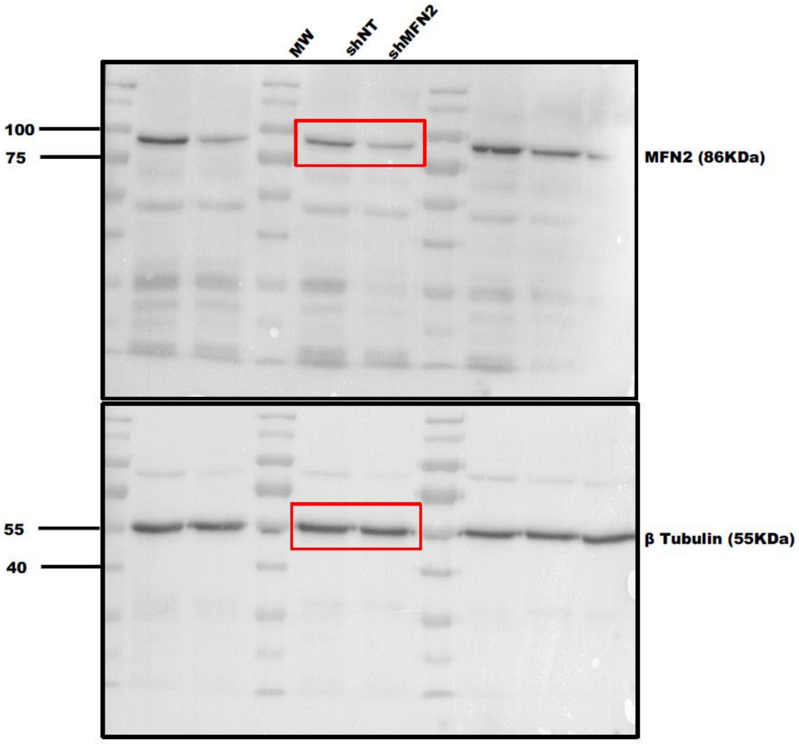

Figure 3A. Ectopic expression of YFP tagged human MFN2 in shMFN2 stable B16 cells

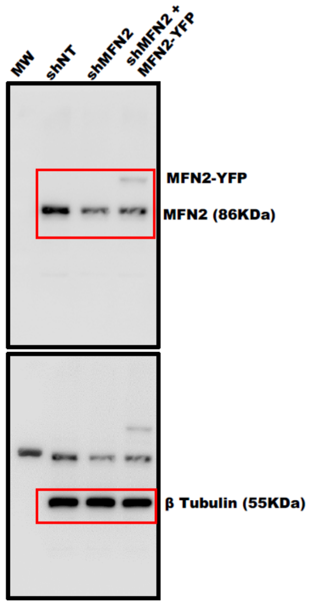

Figure 5A. Protein expression of Gp100 in shNT and shMFN2 stable cells on LD Day7

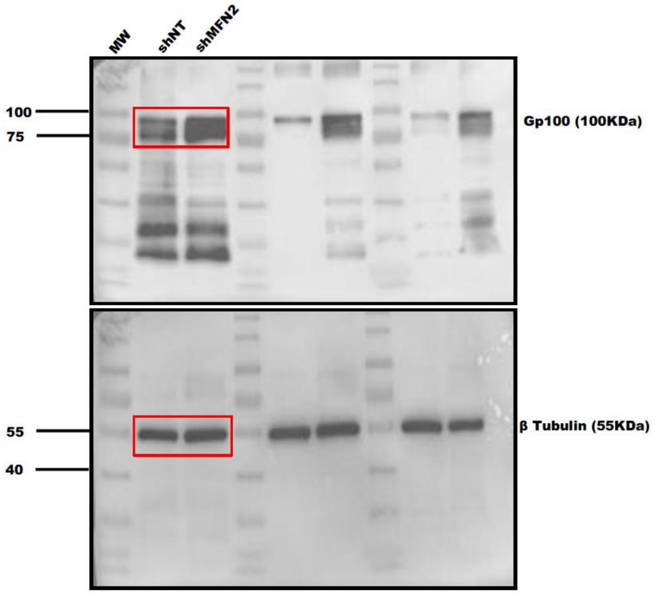

Figure 5B. DOPA assay for tyrosinase enzyme activity and protein expression of tyrosinase in shNT and shMFN2 stable cells

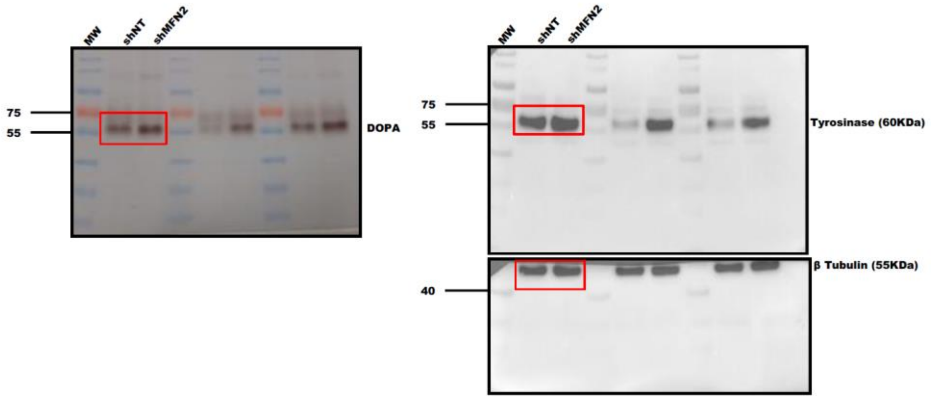

Figure 5C. Protein expression of DCT in shNT and shMFN2 stable cells

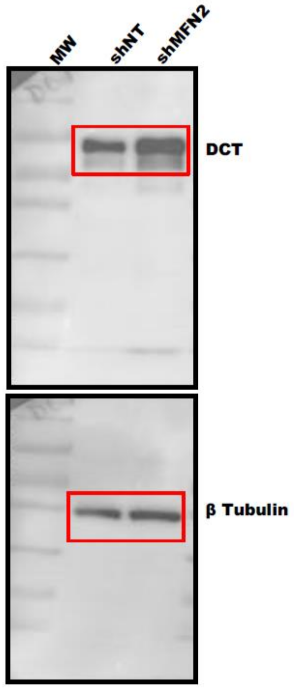

Figure 5D. DOPA assay for tyrosinase enzyme activity and protein expression of tyrosinase in shNT, shMFN2 and shMFN2+ MFN2-YFP cells

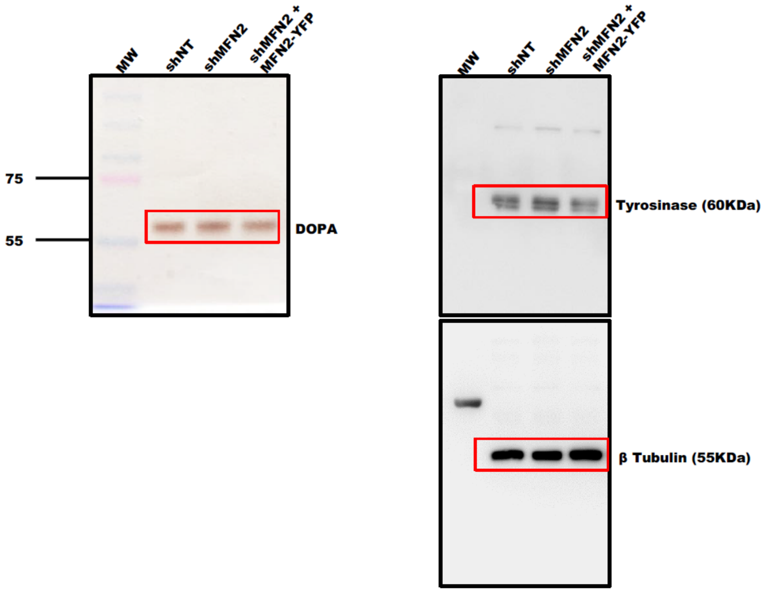

Figure 5E. Protein expression of Gp100 in shNT, shMFN2 and shMFN2+ MFN2-YFP cells

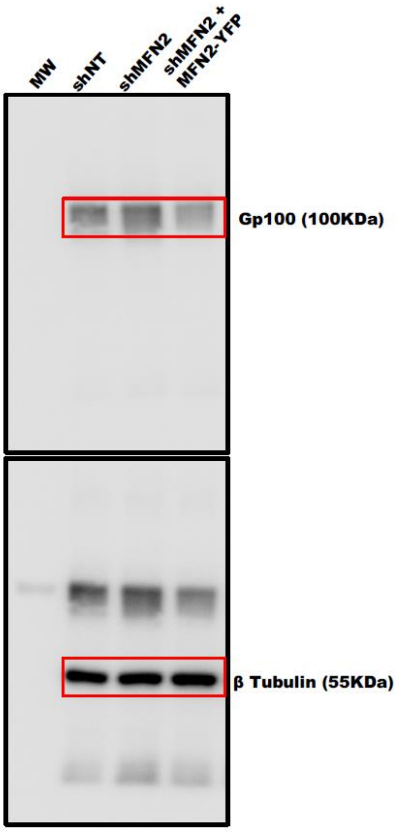

Figure 5F. Protein expression of DCT in shNT, shMFN2 and shMFN2+ MFN2-YFP cells

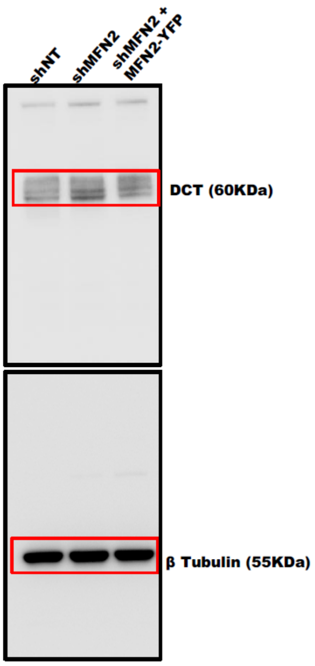

Figure 6A. DRP1 protein expression in LD pigmentation model

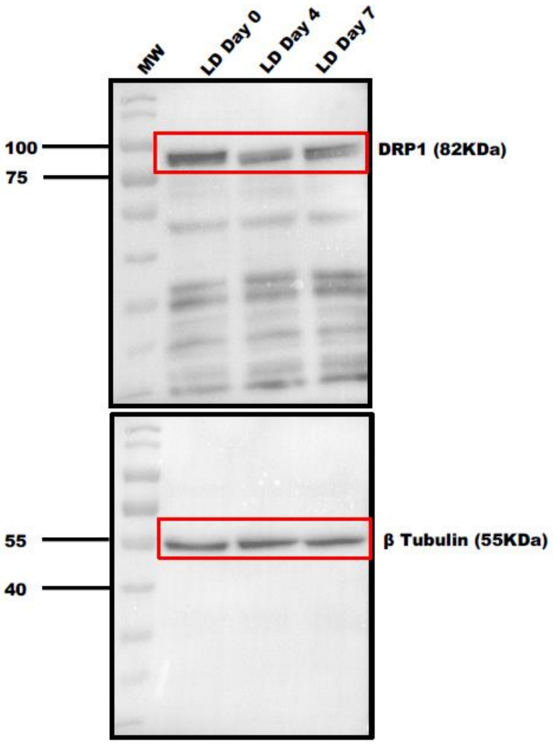

Figure 6C. DRP1 protein expression in LP-DP primary human melanocytes

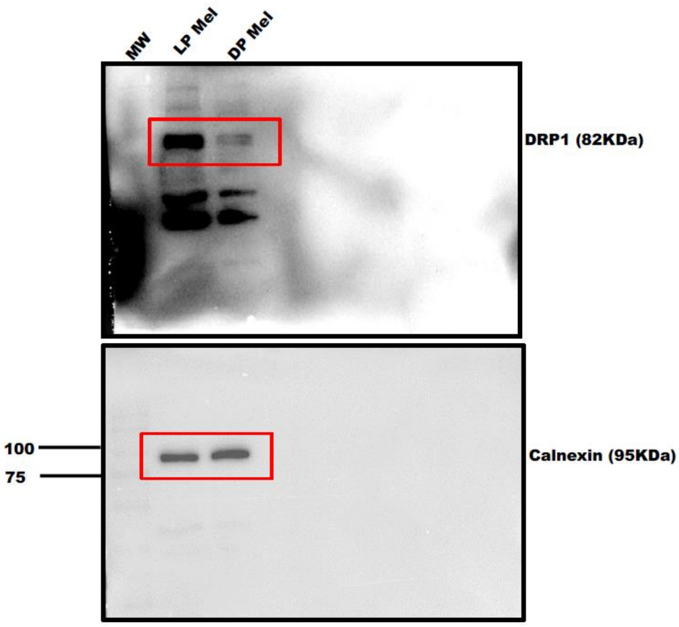

Figure 6E. MFN1 protein expression in LD pigmentation model

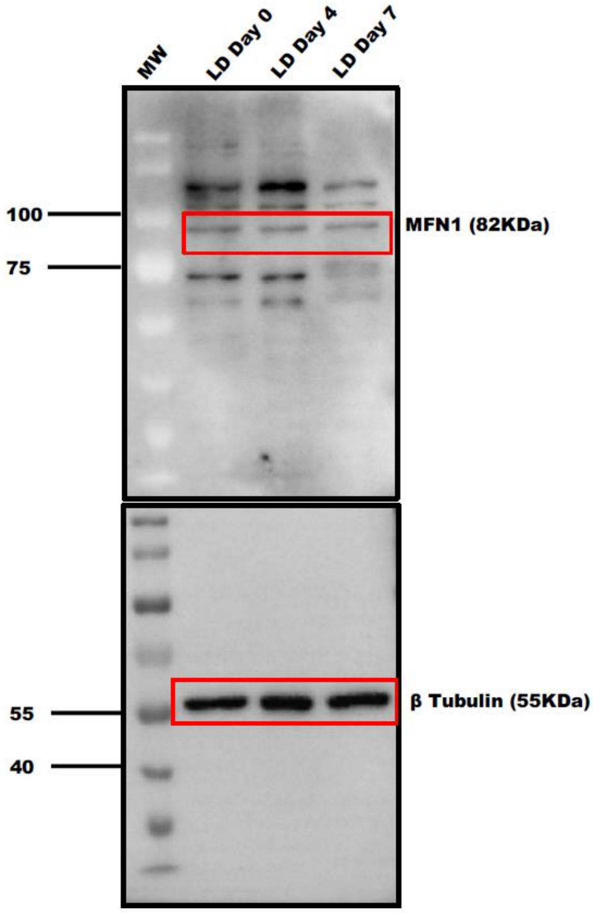

Figure 6G. MFN1 protein expression in LP-DP primary human melanocytes

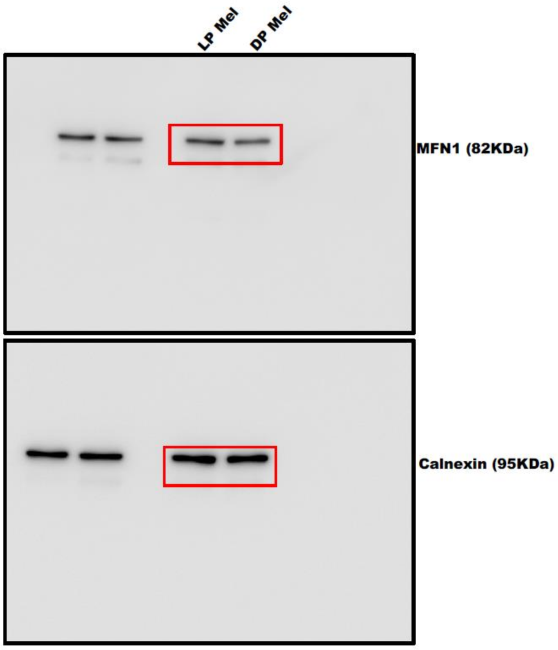

Figure 7A. siRNA based silencing of DRP1 in B16 cells

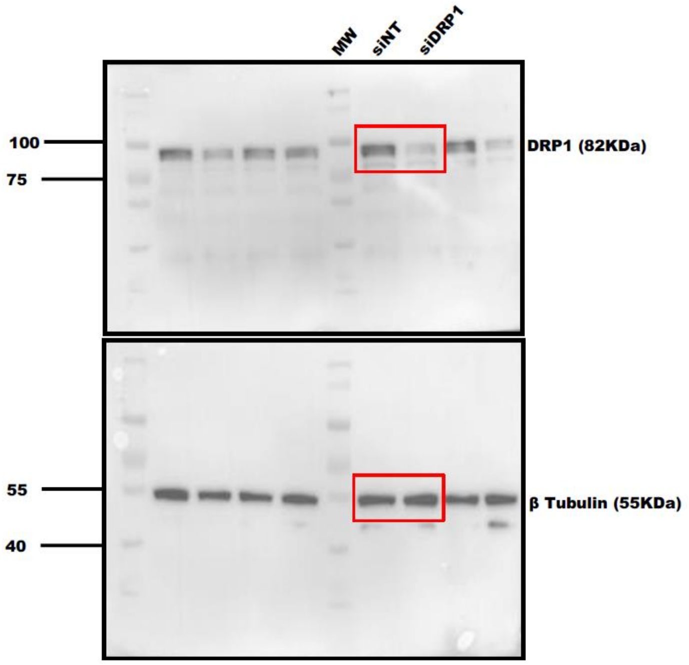

Figure 7C. shRNA mediated knockdown of DRP1 in B16 cells

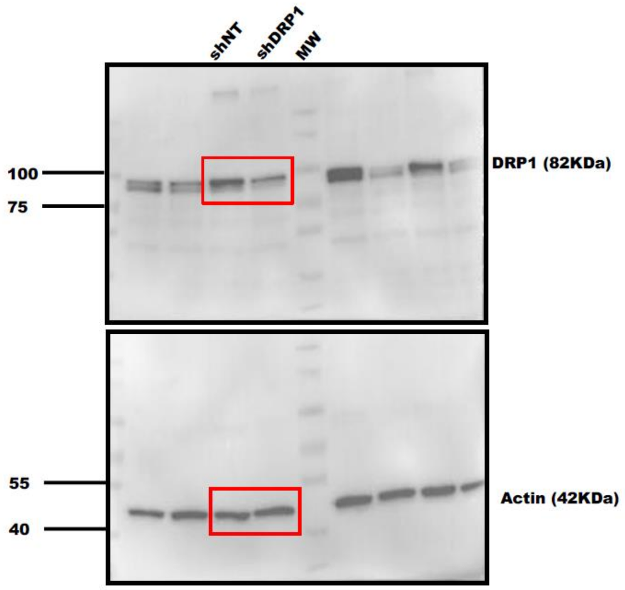

Supplement: Supplementary file 1 [file cells-11-00701-s001.zip › cells-1572253 sup/cells-1572253 sup .pdf]
