# Supplementary material for: Mitofusin-2 Negatively Regulates Melanogenesis by Modulating Mitochondrial ROS Generation
Source: Cells. 2022 Feb 16;11(4):701. doi: 10.3390/cells11040701 (PMC8869806; doi:10.3390/cells11040701)
Supplement: Supplementary file 1 [file cells-11-00701-s001.zip › cells-1572253 sup/cells-1572253 sup 2.pdf]

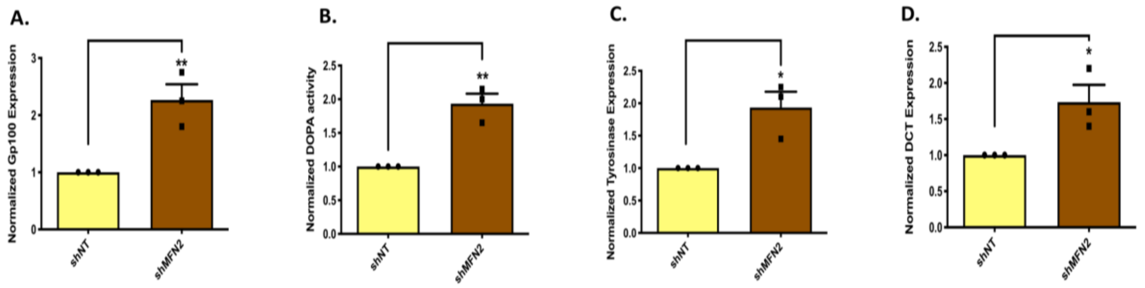

Supplementary Figure 1. MFN2 regulates melanogenic enzyme expression and activity. A. Quantitation of densitometric analysis of Gp100 expression in shNT and shMFN2 cells on LD Day7 (N = 3). **B.** Quantitative analysis of tyrosinase activity in shNT and shMFN2 cells on LD Day7 (N = 3). **C.** Quantitation of densitometric analysis of tyrosinase expression in shNT and shMFN2 cells on LD Day7 (N = 3). **D.** Densitometric analysis of DCT expression in shNT and shMFN2 cells on LD Day7 (N = 3). Data presented are Mean ± S.E.M. (* $p < 0.05$; ** $p < 0.01$; Unpaired Student's *t*-test was performed for statistical analysis).
